# Faut-il continuer à infiltrer le scalp par un anesthésique local pour une craniotomie?

**DOI:** 10.11604/pamj.2015.22.2.6309

**Published:** 2015-09-03

**Authors:** Mouhssine Doumiri, Youssef Motiaa, Rachid Razine, Morad Amor, Abdelmajid Moussaoui, Saad Kabbaj, Wajdi Maazouzi

**Affiliations:** 1 Service d'Anesthésie-réanimation, Hôpital des Spécialités, Centre Hospitalier Universitaire, Université Mohammed V, Rabat, Maroc; 2 Laboratoire de Santé Publique, de Biostatistique, Recherche Clinique et Epidémiologie, Faculté de Médecine et de Pharmacie de Rabat, Rabat, Maroc

**Keywords:** Scalp, infiltration, lidocaïne, anesthesie local, craniotomie, Scalp, infiltration, lidocaine, local anesthesia, craniotomy

## Abstract

**Introduction:**

Le maintien de la stabilité hémodynamique est un objectif primordial de l'anesthésie pour craniotomie. Peu d’études ont prouvé l'efficacité de l'infiltration du scalp par un anesthésique local pour le maintien de la stabilité hémodynamique après l'incision. L'objectif de notre travail est d’évaluer l'influence de l'infiltration de la ligne d'incision du scalp par la lidocaïne à 0,5% adrénalinée sur les paramètres hémodynamiques après incision pour craniotomie supratentorielle.

**Méthodes:**

Étude prospective en double aveugle réalisée au bloc opératoire de l'hôpital des spécialités de Rabat sur une période d'une année incluant 80 malades programmés pour craniotomie supratentorielle. Les malades étaient randomisés par tirage au sort en 2 groupes: Le groupe 1 était infiltré par 40 ml du sérum salé adrénaliné (1/200 000) et le groupe 2 était infiltré par 40 ml de la lidocaïne 0,5% adrénalinée (1/200 000). Le critère de jugement principal était la pression artérielle moyenne (PAM) après l'incision. L’étude statistique a fait appel aux tests t de student et l'U- mann-whitney. Une valeur de p <0.05 était considérée significative.

**Résultats:**

L’étude a inclus 80 patients (40 hommes et 40 femmes). L’âge moyen était 42,33±14,76 ans. Le poids moyen était 71,58 ±10 kg. Le 3/4 des patients était ASA 1, seulement 25% étaient ASA2. La durée moyenne de la chirurgie était de 252,06±38,62 mn. Les deux groupes étaient comparables concernant l’âge, le sexe, le poids, la durée d'intervention, le type d'abord chirurgical, la dose totale du fentanyl reçue jusqu'a l'incision, ainsi que les paramètres hémodynamiques avant l'incision. Après l'incision la FC moyenne a augmenté dans les deux groupes: 80,53±7,72 bpm dans le groupe contrôle et 76,85±8,52 bpm dans le groupe lidocaïne. La différence d'augmentation de la FC entre les deux groupes était statistiquement significative (p = 0,047). L'augmentation de la PAM était également significativement plus élevée dans le groupe placebo (96,45± 3,53mmHg vs 94,75± 3,76mmHg) (p = 0,041). Nous n'avons pas noté de troubles de rythme ou d'hypertension artérielle par les solutions adrénalinées à 1/200000. Par contre, six cas d'hypotension artérielle ont été notés (3cas dans chaque groupe) après 2 minutes de l'infiltration et ayant répandu au remplissage par 500 ml de sérum salé 0,9%.

**Conclusion:**

L'infiltration par la lidocaïne procure une stabilité hémodynamique (PAM et FC) statistiquement significative. Les autres études rapportées dans la littérature et avec un échantillon réduit ont permis de retrouver une différence significative concernant uniquement la PAM et non la fréquence cardiaque.

## Introduction

Le maintien de la stabilité hémodynamique est un objectif primordial de l'anesthésie pour une procédure neurochirurgicale. Les stimuli anesthésiques (induction, laryngoscopie et intubation) et les stimuli chirurgicaux (mise en place de la têtière de Mayfield, incision cutanée, mise en mise en place des agrafes, craniotomie et ouverture de la dure-mère) s'accompagnent fréquemment d'une tachycardie et d'une hypertension artérielle, malgré une anesthésie générale adéquate et bien conduite [1, 2]. Ces modifications hémodynamiques peuvent être responsables chez les malades porteurs d'anomalies vasculaires intracérébrales (anévrysmes, malformations artério-veineuses) ou ceux dont l'autorégulation cérébrale est altérée, d'une augmentation brutale du débit sanguin cérébral et de la pression intracrânienne. Plusieurs méthodes sont proposées pour faire face aux modifications hémodynamiques liées aux stimulations douloureuses peropératoires: prémédication (clonidine [3], gabapentin [4]…), approfondissement de l'anesthésie et réinjections des analgésiques [5], bloc nerveux du scalp ou infiltration du scalp par un anesthésique local [6–8], médicaments comme alpha ou bétabloquants et lidocaine intraveineux [9–11]. L'objectif de notre travail est d’évaluer l'influence de l'infiltration de la ligne d'incision du scalp par la lidocaîne à 0,5% adrénalinée sur les paramètres hémodynamiques après incision pour craniotomie supratentorielle.

## Méthodes

**Figure 1:**
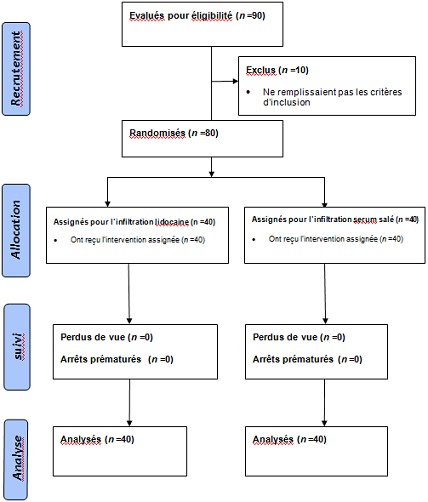
Diagramme consort

Après consentement des malades et accord du comité d’éthique local de l'hôpital des spécialités de rabat( spécialisé en anesthésie- réanimation, neurochirurgie, neurologie, ophtalmologie et chirurgie oto-rhino-laryngologie), 80 malades ASA 1 et 2 (American Society of Anesthesiologists), devant subir une craniotomie programmée pour une tumeur supratentorielle ont été inclus dans cette étude prospective randomisée en double aveugle réalisée au bloc opératoire de l'hôpital des spécialités de Rabat sur une période d'une année (du premier janvier 2011 jusqu'au 31 décembre 2011). Les malades qui avaient une hypertension artérielle, une cardiopathie sous jacente, antécédent de craniotomie, allergie pour certaines drogues, porteurs d'une pathologie anévrysmale cérébrale ou sous bêta bloquant étaient exclus de l’étude (Figure 1). Les malades n’étaient pas prémédiqués. Le monitorage péropératoire (datex) comportait un électro cardioscope avec cinq électrodes, un tensiomètre automatique pour mesure de la pression non invasive et invasive, un saturomètre et un capnographe. L'anesthésie était induite par du fentanyl 5µg/kg, du thiopental 8mg/kg et du vecuronuim 0.1m/kg en IV. La lidocaîne à la dose de 1,5mg/kg en IV était administrée à tous les malades avant la laryngoscopie. Après l'intubation endotrachéale, l'anesthésie était entretenue par des réinjections de thiopental et de fentanyl jusqu’à l'ouverture de la dure-mère, puis l'isoflurane, sans protoxyde d'azote, jusqu’à la fin d'intervention. Des seringues de 50 ml pour infiltration étaient préalablement préparées et numérotées en G1 (groupe sérum salé) ou G2 (groupe lidocaîne). L'anesthésiste de la salle opératoire et le chirurgien n’étaient pas informés sur le groupe de la seringue utilisé pour l'infiltration. Après position chirurgicale du malade et application des champs, la surface chirurgicale était stérilisée. Les malades étaient randomisés par ordinateur en deux groupes. Le groupe 1 était infiltré par 40 ml de sérum salé physiologique adrénaliné à (1/200000) et le groupe 2 était infiltré par 40 ml de lidocaîne 0,5% adrénalinée à (1/200000). Une dose de 1,5µg/kg de fentanyl en IV était administrée pour les deux groupes 5min avant l'incision. La fréquence cardiaque (FC), la pression artérielle systolique (PAS), la pression artérielle diastolique (PAD) et la pression artérielle moyenne (PAM) avant et après l'incision et toute les 3 minutes jusqu’à l'ouverture de la dure-mère étaient relevées et comparées. Après l'infiltration du scalp, une durée de 5 minutes était respectée avant l'incision. Le type d'incision et la dose moyenne de fentanyl étaient également notés et comparés chez les deux groupes. Le critère de jugement principal était la PAM avant et après l'incision. Le choix de la lidocaîne par rapport à un autre anesthésique était essentiellement du a la disponibilité du produit et à la limitation de l’étude aux variations hémodynamiques au premier temps chirurgical. Le traitement des pics hypertensifs (pression artérielle supérieur à 20% des valeurs de base) a fait appel à la nicardipine par titration de 0,5 mg en IV. L’étude statistique a fait appel aux tests t de Student et Chi carré et aux tests non paramétrique de Kruskal-Walliss et le U- Mann-Whitney. Une valeur de p < 0.05 était considérée comme significative.

## Résultats

L’étude a inclus 80 patients (40 hommes et 40 femmes) avec un âge moyen de 42,33±14,76 ans (extrêmes15-70 ans). Le poids moyen était de 71,58±10 kg (extrêmes 50 - 90 kg). Les 3/4 des patients étaient ASA 1 et seulement 25% étaient ASA 2 (essentiellement des diabétiques). La durée moyenne de la chirurgie était de 252,06±38,62min (extrêmes 180- 330 min). Les deux groupes étaient comparables concernant l’âge, le sexe, le poids, la durée d'intervention, la dose totale de fentanyl reçue jusqu’à l'incision ainsi que les types d'incisions pratiquées (Tableau 1) et les paramètres hémodynamiques avant l'incision (Tableau 2). Après l'incision la FC moyenne a augmenté dans les deux groupes: 80,53±7,72bpm dans le groupe contrôle et 76,85±8,52bpm dans le groupe lidocaïne. La différence d'augmentation de la FC entre les deux groupes était statistiquement significative (p = 0,047). L'augmentation de la PAM était également significativement plus élevée dans le groupe placebo (96,45± 3,53mmHg vs 94,75± 3,76mmHg) (p = 0,041) (Tableau 3). Nous n'avons pas noté de troubles de rythme ou d'hypertension artérielle par les solutions adrénalinées à 1/200000. Par contre, six cas d'hypotension artérielle ont été notés (3 cas dans chaque groupe) après 2 minutes de l'infiltration et ayant répandu au remplissage par 500 ml de sérum salé 0,9%.

**Tableau 1:** Comparaison des variables démographiques

Variables	Groupe 1 Placebo	Groupe 2 lidocaïne	p
**Age moyen**	43,10±14,9	41,55±14,7	0,642
**ASA**			1
ASA I	30 (75%)	30 (75%)	
ASAII	10 (25%)	10 (25%)	
**Sexe**			0,823
Hommes	22 (55%)	21 (47, 5%)	
Femmes	18 (45%)	19 (22, 5%)	
**Poids (kg)**	72,15±10,54	71±9,65	0,612
**Durée d'intervention(min)**	248,18±52,36	251±39,92	0,787
**Dose de fentanyl (µg/kg) jusqu’à l'incision**	8	8	1
**Types d'incision:**			0,69
Sous-frontale	13 (32,5%)	15(37, 5%)	
Fronto-temporale	10 (25%)	10(25%)	
Fronto-pariétale	6 (15%)	5(12,5%)	
Pariéto-temporale	11 (27, 5%)	10(25%)	

**Tableau 2:** Comparaison des variables hémodynamiques en pré-incision

Variable	Groupe 1	Groupe 2	p
FC (bpm)	71,85±8	70,6±6,96	0,459
PAS (mmHg)	118,8±6,45	119,67±6,45	0,546
PAD (mmHg)	69,97±3,83	70,28±3,99	0,732
PAM (mmHg)	87,6± 3,96	88,28± 3,92	0,441

**Tableau 3:** Comparaison des variables hémodynamiques en post-incision

Variable	Groupe 1	Groupe 2	p
FC (bpm)	80,53±7,72	76,85±8,52	0,047*
PAS (mmHg)	129,02±7,66	125,75±7,74	0,061
PAD (mmHg)	72,72±3,49	72,82±3,53	0,899
PAM (mmHg)	96,45± 3,53	94,75± 3,76	0,041*

## Discussion

L'incision du scalp au cours d'une craniotomie est associe a des modifications hémodynamiques importantes même sous anesthésie générale qui peuvent contribuer à l'augmentation de la pression intracrânienne. Plusieurs techniques ont été utilisées pour les atténuer: (1) L'administration des doses élevées des opioïdes, souvent efficace, peut être responsable d'une dépression respiratoire prolongée postopératoire nécessitant une ventilation artificielle prolongée, empêchant une évaluation neurologique immédiate à la sortie de la salle opératoire et augmentant la durée de séjour à la salle de surveillance post interventionnelle (SSPI). (2) La réalisation d'un bloc nerveux au niveau du scalp par un anesthésique local [7]. (3) L'infiltration du scalp par un anesthésique locale. Cette dernière est une pratique courante et ancienne mais peu d’études ont évalué l'impact de l'infiltration par un anesthésique local sur les variations hémodynamiques de l'incision. Notre série montre que l'infiltration par 40 mL de la lidocaine à 0,5% adrénalinéé 1/200000 à permis d'atténuer l'augmentation de la PAM et la FC après l'incision du scalp. Le choix de la lidocaine dans notre série était basé sur sa disponibilité dans notre formation et que l’étude était limitée à la phase initiale de la craniotomie (de l'induction de l'anesthésie jusqu’ à l'ouverture de la dure mère). L'infiltration du scalp par La lidocaïne à 1% non adrénalinée avec une dose moyenne de (9,9 ± 1,95 ml) a été évaluée dans l’étude de Pakulski et all [8] ou 100 patients étaient choisis et divisés en 4 groupes de 25 patients: le groupe IA (tumeur cérébrale, sans infiltration), le groupe I B (anévrysme cérébrale, sans infiltration) le groupe II A (tumeur cérébrale avec infiltration), le groupe II B (anévrysme cérébrale avec infiltration). L'infiltration du scalpe a permis d'atténuer de façon significative la PAM après l'incision du scalp mais sans avantage sur la fréquence cardiaque. L'infiltration du scalp par la bupivacaine, associée ou non à l'adrénaline, a été aussi évaluée. Elle a montré son efficacité pour atténuer la réponse hémodynamique de l'incision du scalp. Dans l’étude randomisée en double aveugle de Bloomfield et all [2], 36 adultes ASA 1 et 2 devant subir une craniotomie programmée ont été répartis en deux groupes: un groupe était infiltré par 0,42 ± 0,15 ml/kg de bupivacaine à 0,25% adrénalinée à 1/200000 et un groupe était infiltré par 0,47 ± 0,17 ml/kg de sérum salé adrénaliné à 1/200000. Cette étude a mis en évidence que la PAM montait significativement après incision cutanée dans le groupe sérum salé alors que la fréquence cardiaque restait stable dans les deux groupes. Engberg et al [12], dans une étude prospective randomisée en double aveugle portant sur 10 malades par groupe, ont montré que l'augmentation de la PAM était significativement plus élevée dans le groupe infiltré par sérum salé par rapport au groupe infiltré par bupivacaine à 0,25% (P< 0.0005). Hillman et al [1] dans une étude prospective randomisée (21 par groupe) a montré l'efficacité de l'infiltration du scalp par bupivacaine 0,5% mL non adrénalinée avec une dose de 16,4 ± 5ml dans la réduction de la PAM et la Fc après l'incision cutanée en comparaison avec un groupe placebo. Le but d'utilisation d'une solution d'anesthésique local adrénaliné dans notre série était basée sur l'effet vasoconstricteur de l'adrénaline en diminuant le saignement du cuir chevelu, la distribution de l'anesthésique local et par conséquence sa toxicité systémique.

Nous avons noté, 3 cas d'hypotension par groupe après deux minute de l'infiltration alors que nos patients était hemodynamiquement stable avant et au cours de l'infiltration de telles épisodes ont été notée dans d'autres séries. Murthy et all [13] ont utilisé pour l'infiltration du scalp chez 112 patients programmés pour craniotomie 5 solutions adrénalinées à différentes concentrations. Le groupe A (lidocaïne 0,5%), le groupe B (lidocaîne avec adrénaline à 1/200000), le groupe C (lidocaïne avec adrénaline à 1/100000), le groupe D (sérum physiologique avec adrénaline à 1/200000) et le groupe E (sérum physiologique avec adrénaline à 1/100000). Des épisodes de tachycardie se sont produits plus fréquemment dans le groupe E (P = 0.03) alors que la lidocaïne seule n'a pas causé de changement significatif de la tension artérielle. L'incidence de l'hypertension artérielle systolique, diastolique, et moyenne était accrue de manière significative dans le groupe E (P= 0.01). Des épisodes d'hypertension diastolique se sont produits plus fréquemment dans le groupe D (P= 0.01). Une hypotension artérielle diastolique et moyenne biphasée (autour de minute 2 et de minutes 9-15) a été notée dans les groupes B et C (P = 0.001) [13]. Dans l’étude de Yang et al [14], 120 patients, subissant une craniotomie programmée, étaient randomisés en 4 groupes. Tous les patients ont reçu 16mL de la lidocaïne à 1% avec adrénaline à différentes concentrations: le groupe 1 avec 40 µg (2.5 mg/ml); le groupe 2 avec 80µg (5mg/mL); le groupe 3 avec 160 µg (10 mg/ml); et le groupe 4 (groupe de control) sans adrénaline. La pression artérielle moyenne et la fréquence cardiaque étaient mesurées toutes les 30 secondes pendant 5 minutes après l'infiltration. La diminution de la PAM et l'augmentation de la fréquence cardiaque à 1,5 minute après le début de l'infiltration locale étaient observées dans le groupe 1, le groupe 2 et le groupe 3 (P < 0.001) mais pas dans le groupe 4. L'augmentation de la PAM n’était notée que dans le groupe 3. Ces hypotensions par de faibles concentrations d'adrénaline sont expliquées par une stimulation des récepteurs bêta 2 adrénergiques qui sont responsables d'une vasodilatation et d'une hypotension.

## Conclusion

Cette étude a été réalisée pour vérifier le bien-fondé de l'infiltration des anesthésiques locaux dans la modulation des variables hémodynamiques liés à l'incision. D'après les résultats de cette série, l'infiltration par la lidocaïne procure une stabilité hémodynamique (PAM et FC) statistiquement significative. Les autres études rapportées dans la littérature et avec un échantillon réduit ont permis de retrouver une différence significative concernant uniquement la PAM et non la fréquence cardiaque. D'autres études à grande échelle sont nécessaires.
